# Reads like a Novel

**Published:** 1903-07

**Authors:** 


READS LIKE A NOVEL.—Dr. Jno. Punton, the talented editor of the *Kansas City Medical Index Lancet,* and an expert alienist and neurologist, in the July number of his journal writes up the now famous case of Oran Hoskins, who claimed to have been "knocked silly" by a grain door and whose mother sued the St. Louis and San Francisco Railroad Company for $75,000, getting a judgment for $35,000. Hoskins played it upon the doctors successfully for nine months. Dr. Punton was sent to Fort Worth to examine the case. He said Hoskins was *malingering,* and was laughed at; but he finally induced Dr. Walker, who had the case in hand, to take his view of the case. Walker put Hoskins through a series of tests and caught him out. Judgment reversed, and Hoskins and mother indicted for conspiracy to defraud railroad company.

